# Cerebral Oxygen Delivery and Consumption in Brain-Injured Patients

**DOI:** 10.3390/jpm12111763

**Published:** 2022-10-25

**Authors:** Dorota Siwicka-Gieroba, Chiara Robba, Jakub Gołacki, Rafael Badenes, Wojciech Dabrowski

**Affiliations:** 1 Department of Anaesthesiology and Intensive Care, Medical University in Lublin, 20-954 Lublin, Poland; 2 Department of Anesthesiology and Intensive Care, San Martino Policlinico Hospital, IRCCS for Oncology and Neurosciences, 16132 Genoa, Italy; 3 Department of Surgical Sciences and Integrated Diagnostics (DISC), University of Genoa, 16132 Genoa, Italy; 4 Department of Anesthesiology and Surgical-Trauma Intensive Care, Hospital Clinic Universitari, University of Valencia, 46010 Valencia, Spain

**Keywords:** brain, oxygen, delivery, consumption, TBI

## Abstract

Organism survival depends on oxygen delivery and utilization to maintain the balance of energy and toxic oxidants production. This regulation is crucial to the brain, especially after acute injuries. Secondary insults after brain damage may include impaired cerebral metabolism, ischemia, intracranial hypertension and oxygen concentration disturbances such as hypoxia or hyperoxia. Recent data highlight the important role of clinical protocols in improving oxygen delivery and resulting in lower mortality in brain-injured patients. Clinical protocols guide the rules for oxygen supplementation based on physiological processes such as elevation of oxygen supply (by mean arterial pressure (MAP) and intracranial pressure (ICP) modulation, cerebral vasoreactivity, oxygen capacity) and reduction of oxygen demand (by pharmacological sedation and coma or hypothermia). The aim of this review is to discuss oxygen metabolism in the brain under different conditions.

## 1. Introduction

Organism survival depends on oxygen delivery and utilization to maintain the balance of energy and toxic oxidants production [1]. This regulation is crucial to the central nervous system (CNS). Brain tissue presents a peculiarly dynamic consumption of energy. The most productive metabolic process of energy analogs is oxidative phosphorylation, relating to oxygen consumption [2]. Already in 1890, Roy and Sherrington observed that increased neuronal activity elevates energy consumption and compensatory metabolic and vasculature reactions, which in turn improve the functionality of neurons [3]. Therefore, the oxygen level in cerebral tissue is a crucial element that impacts nerve and glial cell functions [2].

Brain injury is a common cause of morbidity and mortality worldwide, especially in the young population [4]. Secondary brain damage occurs in the hours, days or weeks after an event, and is associated with fatal outcomes [5]. Secondary insults may be mediated by impaired cerebral metabolism, ischemia, intracranial hypertension and oxygen concentration disturbances such as hypoxia [6,7]. The combination of hypoxia and hypotension is associated with enormously high mortality rates [8]. Recent data highlight the important role of clinical protocols in improving oxygen delivery and resulting in lower mortality in traumatic and nontraumatic brain-injured patients [9,10]. Clinical protocols guide the rules for oxygen supplementation based on physiological processes such as increased oxygen supply (by monitoring of mean arterial pressure (MAP) and intracranial pressure (ICP), cerebral vasoreactivity and oxygen capacity) and reduction of oxygen demand (by pharmacological sedation and coma or hypothermia) [11]. Therefore, monitoring oxygen concentrations such as brain tissue oxygen (PbtO_2_) is an important aspect of brain injury clinical practice [12]. In addition, monitoring of mean arterial pressure and oxygenation of both local and global tissues are essential for oxygenation and final outcomes [13].

The aim of this review is to discuss oxygen metabolism in the brain under different conditions.

## 2. Oxygen Delivery and Autoregulation

The weight of the brain is only 2% of the human body, but cerebral tissue uses 25% of the glucose and about 20% of the oxygen delivered to function normally [14]. Oxygen consumption is 3.5 mL of oxygen/100 g tissue/1 min; therefore, the regulation of blood flow and delivery of oxygen to cerebral tissue is crucial for brain function [15]. Importantly, 75–80% of the energy consumed by neurons is used at the synapses to restore the neuronal membrane potentials lost during depolarization [16]. The continuous supply of oxygen to the brain occurs via arterial blood and is transported to brain tissue by diffusion. Diffusion is linked to the oxygen conductivity of cerebral tissue, determined by the geometry of capillaries (distance and area) and the metabolism of tissue (oxygen gradient from capillary to tissue) [17]. Extraction of oxygen is inversely proportional to blood flow (when metabolism is constant) and directly proportional to metabolism (when flow is constant) and the area between tissue and capillaries. Thus, a reduction in oxygen delivery increases oxygen extraction. It should be noted that when cerebral blood flow (CBF) is reduced by 50–60%, the consequent elevation of oxygen extraction is insufficient to maintain proper cerebral oxygenation and a constant cerebral metabolic rate of oxygen (CMRO2) [18]. Thus, cerebral oxygen delivery is determined by blood oxygen content and cerebral blood flow. In physiological conditions, total blood flow in the brain is constant because of the contribution of the large arteries to vascular resistance, as well as the impact of the parenchymal arterioles on considerable basal tone.

Autoregulation of cerebral blood flow is the mechanism that enables the brain to maintain relatively constant blood flow through changes in perfusion pressure [19]. In a normotensive, physiological state, the ensuing cerebral perfusion pressure (CPP) is in the range of 60 to 160 mmHg, and CBF is maintained at 50 mL per 100 g of brain tissue per minute. Outside of this range, autoregulation is lost, and CBF starts to be dependent on MAP in a linear mode [20]. A drop of CPP below the lower limit of 50 mmHg results in cerebral ischemia [21]. This reduction of CBF is compensated for by elevated oxygen extraction from the blood.

The individualization of care by targeting optimal, near to cerebral autoregulation (CA)-guided CPP is connected with improved outcomes in TBI patients [22]. It is worth remembering that combined brain tissue oxygen with ICP/CCP-guided therapy strongly ameliorates favorable long-term outcomes [23]. In addition, in a recent meta-analysis, Xie et al. documented that this combined therapy did not present any effects on mortality, ICP/CPP and length of stay of patients after TBI [23].

Over a physiological range of partial oxygen pressure (PaO_2_) (75–100 mmHg; 7–13.33 kPa), PaO_2_ has little effect on global CBF as long as it does not fall below 50 mmHg (6.67 kPa). This is because CBF is connected to the arterial content of oxygen rather than PaO_2_. The form of the hemoglobin–oxygen dissociation curve indicates that the arterial content of oxygen is comparatively stable over the discussed PaO_2_ range [24].

The primary gradient determining the oxygen level in the brain may be enhanced by a gradient-independent mechanism of cerebral vessel tone changes and increases in CBF during functional neural activation (neurovascular coupling) [25,26]. The main role of this mechanism is to transport higher levels of oxygen in advance of the elevated consumption during neuronal activation [27].

Impairment of cerebral perfusion and metabolism following brain injury has been documented repeatedly. Unfavorable outcomes after brain injury are connected with hypoperfusion and decreased glucose metabolism and CMRO_2_ [28]. Recent data have documented a connection between CMRO_2_ and Glasgow Coma Score (GCS) after traumatic brain injury [29,30,31,32]. Soustiel et al. demonstrated that in TBI patients, CBF is somewhat reduced during the first 24 h, and greater hypovolemia is observed following poor outcomes. Importantly, a decrease in CMRO_2_ and the cerebral rate of glucose metabolism (CMRG) correlates with worse outcomes [32].

## 3. Oxygen Consumption

Oxygen is transported to the cerebral cells by blood diffusion from the capillary to the mitochondria, until it is consumed in the mitochondria as part of oxidative metabolism. CMRO_2_ is the rate of consumption and energy homeostasis in the brain and in healthy, awake people, averages 3.3 mL/100 g/min [33]. It is related to CBF. Under elevated metabolic demand, the cerebral vasculature dilates to supply an appropriate increase in CBF.

Importantly, with elevated neural activity, CMRO_2_ also rises [34,35]. In a normal, unstimulated brain, energy is mostly provided by glucose oxidation. Nevertheless, the metabolic rates of the oxygen-to-glucose ratio, CMRO_2_/CMR(glc), called the oxygen-to-glucose index (OGI), increase during activation and diverge from the textbook value of 6. In addition, the levels of lactates in the brain increase during sensory (e.g., visual) stimulation [34]. This oxidative metabolism yields more energy as compared to glycolysis, but precise measurements of this process are limited [36]. Mitochondria present a high metabolic activity and a critical role in aerobic energy production, and their main function is the production of adenosine triphosphate (ATP) through oxidative phosphorylation.

Mitochondrial dysfunction is a major factor in the occurrence of cell damage. Successful resuscitation during ischemia/reperfusion demands the reestablishment of aerobic metabolism by reperfusion of oxygenated blood. Mitochondria play a fundamental role as effectors of reperfusion injury. Damage to the organelle impairs oxidative phosphorylation and elimination of cytochrome c in the cytosol. The main mechanisms are oxidative stress and Ca^2+^ overload [36].

Disturbances in oxygen delivery stop electron flow and interrupt the generation of the “proton motive force” important in ATP production mentioned above. Of course, cells may produce ATP anaerobically by glycolysis. However, this process is less effective, insufficient for metabolic demands, and the final products are lactates.

## 4. Oxygen in the Cells

**Figure 1:**
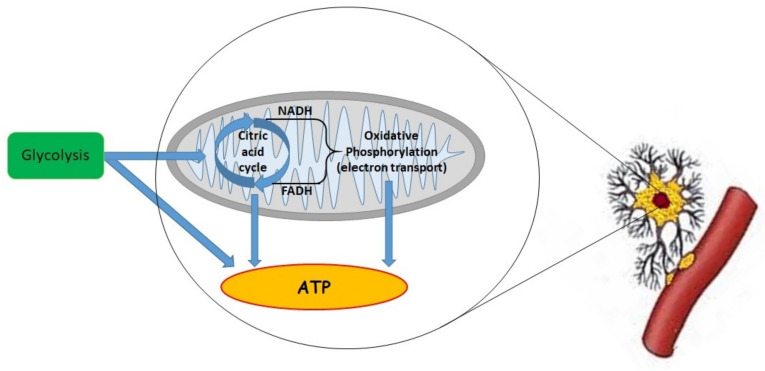
“Mitochondrial respiration” benefits when reducing nicotinamide adenine dinucleotide (NADH) and flavin adenine dinucleotide (FADH_2_) components are created by the tricarboxylic acid (TCA) cycle. In the inner mitochondrial membrane, electrons generated from NADH and FADH2 are oxidized to NAD+ and FAD+ by complexes I and II. Afterward, these electrons are transferred successively to complex III, cytochrome c and complex IV. Cytochrome c oxidase (COX, complex IV) transmits electrons to molecular oxygen. This is an important enzyme in the mitochondrial electron transport chain (ETC) connecting oxygen with oxidative phosphorylation [37]. The transmission of electrons through the ETC is connected with proton transfer from the mitochondrial matrix, across the inner membrane to the intermitochondrial membrane space. This translocation develops an electrochemical gradient of protons (pH gradient and membrane potential). These molecules may drift through the F1Fo-ATP synthase (complex V) or back to the mitochondrial matrix [38]. Importantly, complex V connect protons transfer to the production of ATP from adenosine diphosphate (ADP) and phosphate. Under normal oxygen levels, pyruvate, as a product of glycolysis, is transported into the mitochondria, and is transformed into acetyl-CoA by the pyruvate dehydrogenase (PDH) complex [39]. Afterward, acetyl-CoA connects with oxaloacetate and creates citrate—the first step in the tricarboxylic acid cycle. Reducing equivalents in this cycle impacts ETC to production of ATP and reactive oxygen species (ROS) for signaling, and the intermediates of TCA are used for biosynthetic processes such as lipid synthesis [40].

The role of mitochondria is to maintain maximal levels of ATP in the physiological range of oxygen. It is important to remember that there are also other mechanisms responsible for oxygen consumption. Mostly, O_2_ is consumed by mitochondria, but 1–2% of oxygen is incompletely reduced to superoxide anion (O2−) (Figure 1).

Organisms develop important adaptation mechanisms [1]. One of these mechanisms is metabolic suppression. Reduction of mitochondrial oxygen consumption in cells is observed in oxygen levels between 1 and 3% in vitro. “Oxygen conformance” occurs when the oxygen (<0.3%) level begins to limit the cytochrome c oxidase (COX) (complex IV) [37]. Hypoxia, as a result of limited oxygen accessibility, results in reduction of oxidative phosphorylation and loss of resynthesized phosphates, ATP and phosphocreatine. The ATP-dependent Na/K pump is also changed and promotes the influx of Na, Ca and water into cells, causing cytotoxic edema. In addition, ischemia impacts catabolism of adenine nucleotides, resulting in the accumulation of hypoxanthine in cells. Cytosolic calcium elevation promotes various pathways, such as activation of phospholipases and importantly the release of prostaglandins, lipases, proteases and endonucleases, which damages structural elements of cells [41]. In addition, after increased expression of proinflammatory gene products in the endothelium (leukocyte adhesion molecules, cytokines, endothelin thromboxane A2) a proinflammatory state is observed. In contrast, prostacyclin and nitric oxide are suppressed.

## 5. Hyperventilation/Hypoventilation

One of the most powerful factors affecting cerebral perfusion is hyperventilation/hypoventilation, with an effect on CBF and PaCO_2_. Hyperventilation is a common therapy used to reduce elevated ICP or to relax a tense brain (hypocapnia-reduced CBF and CBV). In traumatic brain injury (TBI) patients, hyperventilation generates a 34% decrease in CBF and a 9% reduction in cerebral blood volume (CBV) when PaCO_2_ is decreased from 40 to 30 mmHg [42]. However, hyperventilation and hypocapnia, apart from vasoconstriction and decreased CBF, also cause neuronal excitability and a longer duration of seizure elevation, an increase in excitatory amino acids and alkalosis of cerebrospinal fluid with a left shift in the oxygen–hemoglobin dissociation curve (OHDC) [43,44]. All these mechanisms may predispose to reduction in oxygen supply and delivery and a significant increase in oxygen extraction.

It should be noted that carbon dioxide is a common molecule with a physiological range of 35–45 mmHg. Hypocapnia (partial pressure of carbon dioxide <35 mmHg) and mild hypercapnia (>45 mmHg) generate important nervous system disturbances. Recent data have documented that hypercapnia presents neuroprotective mechanisms and may improve CBF through cerebral vasodilatation. Hypercapnia also leads to brain edema, elevated ICP, a right shift in the oxyhemoglobin dissociation curve, reduction of systemic vascular resistance (SVR) and an increase in the tissue oxygen availability [45,46].

Hyperventilation is a double-edged sword with some short-term beneficial effects and longer-term risks. The initial PaCO_2_ value in TBI in patients with normal ICP should be within the normal range of 38–42 mmHg. Controlled hyperventilation during mechanical ventilation in TBI patients (never below PaCO_2_ of 30 mmHg) is an approved therapeutic, temporary (during the first 24 h after injury) life-saving intervention in severe intracranial hypertension [46]. However, PaCO_2_ levels should be regulated and individualized in every patient using multimodal neuromonitoring methods [11,47].

## 6. A Brief Search for “4-H” Factors Affecting CRMO2 and Cellular Oxygen Balance

Brain cells are especially susceptible to ischemic damage because of several unusual features of their energy metabolism, high metabolic rate, restricted intrinsic energy stores and critical relationship with the aerobic metabolism of glucose. Therefore, cell metabolism and the consumption of crucial compounds can be altered by drugs or clinical status, which can be summarized as “4-H”.

### 6.1. Hypoxia

The brain is one of the most sensitive organs to hypoxia, reoxygenation and oxidative stress. As mentioned above, the brain has very high metabolic oxygen requirements, and it is highly susceptible to hypoxic damage (Table 1).

Oxidative stress in mitochondria occurs in the state of redox imbalance and small oxidant patterns such as superoxide radical, hydroxyl radical or nitric oxide radicals are accumulated. It should be noted that both hypoxia and hyperoxia may induce oxidative stress and apoptosis [48,49,50]. Acute hypoxia elevates ROS production in the brain, and reoxygenation promotes this process. A low oxygen level leads to increased lipid peroxidation, protein oxidation and nitric oxide levels and antioxidant defense systems. Superoxide dismutase (SOD), reduced glutathione (GSH), glutathione peroxidase (GPx) and reduced/oxidized glutathione (GSH/GSSG) ratio) are significantly inhibited in brain cells. One of the crucial regulators of oxygen homeostasis and angiogenesis control in a hypoxic state are HIFs (hypoxia-inducible factors). There are three transcription factors, HIF-1, HIF-2 and HIF-3 [51]. These heterodimers are expressed by β subunits (HIF-1β, HIF-2β and HIF-3β) and connected with α subunits, HIF-1α, HIF-2α and HIF-3α, directly influencing hypoxia. The HIF-1 and HIF-2 are transcriptional regulators with unique target genes. HIF-1 regulates the acute response to hypoxia (<24 h), and the network formatted by HIF-1 predisposes to elevated perfusion and an increased oxygen level [52].

PaO_2_ has little effect on global CBF as long as it does not fall below 50 mmHg [53]. At this point, there is a dramatic increase in blood flow with a further deterioration in PaO_2_ [53]. Reduction of ATP levels during hypoxia opens K_ATP_ channels on smooth muscle and causes hypopolarization and vasodilatation [54]. Importantly, hypoxia further decreases PaO_2_, and CBF may increase by up to 400% of the baseline level [55]. Changed CBF does not affect metabolism, but hemoglobin saturation decreases from 100% (at PaO_2_ > 70 mmHg (PaO_2_ > 9.33 kPa)) to 50% (at <50 mmHg (at <6.66 kPa)) [55]. In addition, the decrease in PaO_2_ increases the production of local NO and adenosine. Chronic hypoxia increases CBF by affecting capillary density [56,57]. Energy cell failure and delayed apoptosis are connected with NO•, catalyzed by stimulation of nitric oxide synthase (nNOS) by lactic acidosis and disruption of ionic transport [58,59].

In addition, neuronal membrane conversion leads to the release of glutamate, which promotes activation of N-methyl-D-aspartate (NMDA) receptors and calcium influx promoting lipases, proteases and endonucleases, precipitating free radical formation [59,60]. Finally, inflammation, critical mitochondrial dysfunction and ROS (superoxide, hydroxyl, hydrogen peroxide and other) production with oxidation of lipids, proteins, cells and deoxyribonucleic acid (DNA) are observed.

In animal models, oxidative stress parameters and the antioxidant system return to the control system 24 h post brain injury [61]. Coimbra-Costa et al. documented that after 24 h of reoxygenation, oxidative stress is reduced, but apoptosis is preserved, especially in the hippocampus [62]. The apoptotic rate in the hippocampus being higher than in the cortex may be the reason for impairment of brain functions in hypoxic brain damage [63]. One of the crucial regulators of oxygen homeostasis and angiogenesis under a hypoxic state are hypoxia-inducible factors (HIFs) [64]. The HIF-1 binds to hypoxia-responsive elements (HRE) on gene promoters in the nucleus and promotes transcription of target genes such as vascular endothelial growth factor (VEGF), glucose transporter 1 (GLUT1) and others such as glycolysis enzymes, lactate dehydrogenase or erythropoietin [65,66,67]. HIF-1 is also crucial in glycolysis upregulation in astrocytes and Schwann cells [68]. In contrast, HIF-2 and HIF-3 expressions start under chronic hypoxia in the endothelium. Importantly, the switch from HIF-1 to HIF-2 and HIF-3 is observed during the adaptation of the endothelium to prolonged hypoxia. HIF-1 covers the angiogenesis by formation of a primary and very primitive network, and later expression of HIF-2 and HIF-3 stabilizes and promotes maturation of this vasculature [69]. In addition, the network formatted by HIF-1 predisposes to elevated perfusion and increased oxygen level [52].

Another mechanism under chronic hypoxia, which advances proteasomal degradation, is based on the carboxyl terminus of the Hsp70-interacting protein (Hsp70/CHIP complex) [70]. The receptor for activated kinase C1 (RACK1) also leads to degradation of HIF-1α and promotion of heat shock protein 90 (Hsp90), which secures the α subunit [71,72]. Furthermore, RACK1 generates proteasomal degradation and ubiquitination of HIF-1α [73]. Moreover, Kruppel-like factor 2 (KLF2), expressed in endothelial cells and responsible for physiological vascularity formation, activates HIF-1 hypoxic degradation in “a von Hippel–Lindau-independent, but proteasome-dependent manner” via interruption of the connection Hsp90 with HIF-1 [74,75].

MicroRNA (miRNAs) is a family of noncoding RNA molecules with 18–22 nucleotides [76]. There is growing interest in the critical role of miRNA in the development and functioning of the central nervous system as a gene regulator in “cleaving and silencing the gene expression” [77]. In contrast, atypical levels of miRNA are documented in various neurological disorders [78]. The miRNA 210 is mainly expressed in a hypoxic state and is promoted by HIF1 α and establishes a neuroprotective effect in hypoxia–ischemia damage [79,80].

The miRNA molecules decrease the apoptotic processes of neuronal cells with inhibition of caspases [81,82]. Therefore, with the growing interest in the association of miRNA patterns with hypoxia/ischemia, these molecules may be clinical biomarkers for ischemia and an individual miRNA therapeutics complex [83]. The protective effect of glucocorticoids (GCs) under hypoxia and ischemia/reperfusion has been shown recently [84]. GC administration leads to increased tolerance to hypoxia in the central nervous system [85,86]. Acute hypoxia activates hypothalamic–pituitary–adrenal (HPA) with accumulation of up to 24 h of corticosterone in serum [85]. Recent data have shown that hypoxic tolerance is connected with upregulation of HIF-1 α and increased release of GC [85,87]. Direct crosstalk between GC receptors and HIF-1 is potentially a basis of the biochemical pathways for GC upregulation of HIF-1 target genes [88,89,90].

Clinical implications of hypoxia:

- Reduced brain tissue oxygenation is a predictor of poor outcome following severe traumatic brain injury.
- Hypoxic–ischemic brain injury (HIBI) is associated with significant mortality and morbidity [91].
- The LOCO_2_ study documented that targeting lower PaO_2_ improves outcomes in patients with acute respiratory distress syndrome (ARDS) [92].
- The brain tissue oxygen tension (PbtO_2_) is crucial, the second monitored variable after ICP, representing multimodality monitoring in TBI patients [11,93].
- Secondary hypoxia is connected with extended production of cytokines in CSF and superior elevation of serum biomarkers such as myelin-basic protein (MBP) and S100 [94].
- The MBP, S100 and neuron-specific enolase (NSE) biomarkers are more elevated in patients with hypoxia and unfavorable outcomes (Extended Glasgow Outcome Coma Score (GOSE) 1–4) [94]
- HIBI, as a two-hit model, is an effect of primary and secondary ischemic/hypoxic damage predisposing to overall devastating severe injury of neurovascular units [91]
- Secondary brain hypoxia is connected with de novo neuronal and astroglial injury. Importantly, secondary hypoxia is associated with cerebral proinflammatory response but not parallel cerebral endothelial injury [91].
- Protocols based on PbtO_2_ and ICP monitoring significantly decrease cerebral hypoxia time after TBI [95].
- Acute intermittent hypoxia (AIH) and task-specific training (TST) may synergistically improve motor functions after central nervous system injury [96].

### 6.2. Hyperoxia

The concept of hyperoxia toxicity is defined by endogenous production of ROS [48,97].

Experimental examination of mitochondrial structure after 100% oxygen therapy showed swollen and huge mitochondria and diluted and damaged mitochondria membranes and cristae, which were directly connected with myelin, axonal and cellular organelle injury in the cortical brain [98]. Hyperoxia is connected with inhibition of Akt expression and/or phosphorylation, the reverse of low oxygen levels [99,100]. Experimental research has documented that in rat models of hyperoxia (FiO_2_ 0.4–0.8), p-Akt expression decreases steadily, over time until 12 h, then reverses to baseline value [100,101]. Thus, Akt signaling increases in hypoxia and is depressed in hyperoxia [102].

Mitogen-activated protein kinases (MAPKs) are involved in the PI3K-Akt signaling pathway, an important pathway with a neuroprotective role against hypoxia or oxidative stress [103]. Recent data on rat models showed that hyperoxia (FiO_2_ 0.4–0.8) decreases the p-ERK1/2 activation until 12 h and is followed by recovery in the subsequent 12 h [101]. Furthermore, hyperoxia in rat brain models impacts BDNF and neurotrophins 3 and 4 downregulation, and proceeds with correction in the subsequent 15–20 h, predisposing to hyperoxia-linked apoptotic neurodegeneration [101,104]. Erythropoietin receptor (EpoR) binding, observed in different brain areas, also plays an important role in oxygen metabolism [105]. Under hypoxia, it is upregulated because HIF-1α binds to the Epo, showing a neuroprotective effect contrary to ischemia hypoxia/reoxygenation injury [106,107,108]. Experimental data showed that hyperoxia upregulates Epo in mice treated with FiO_2_ 0.5 for 3 weeks, elevating HIF-2α, but during 4 weeks of treatment with FiO_2_ = 0.3, only EpoR expression increases [100]. Noteworthy is NO, which ameliorates oxygen delivery by improving cerebral blood flow in the microvasculature [109]. In addition, NOS presents a neuroprotective effect by improving vessel autoregulation [110]. It triggers different mechanisms such as BDNF expression, HIF stabilization, S-nitrosylation of the HIF, blocking HIF-1α degradation, interaction with MAPK and phosphoinositide 3-kinase (PI3K) signaling, and EpoR expression upregulation [111,112,113,114,115,116]. In a nonphysiological state presenting hyperactivity of selected NOS, the NO starts to be neurotoxic as a free radical [109]. High oxygen concentration controls NOS expression and inhibits NO via surplus release of superoxide anions inhibiting NO vasorelaxation and promoting vasoconstriction in the brain [117,118]. Importantly, the connection of superoxide anions with NO promotes peroxynitrite (ONOO−) production with destructive properties [119,120,121]. In animal research, NO is connected with hyperoxia-induced proliferation and proinflammatory responses in astrocytes via cyclooxygenase-2 and prostaglandin E2 suppression [122].

Clinical implications of hyperoxia:

- Hyperoxia is associated with higher mortality and worse short-term functional outcomes, especially in patients who receive uncontrolled oxygen delivery during the first 24 h after brain injury (probably because of hyperoxia-induced oxygen-free radical toxicity with or without vasoconstriction) [123].
- Potential toxicity of a high oxygen concentration (patients receiving FiO_2_ of more than 0.6).
- Previous studies documented that higher inspired oxygen concentration is associated with acute lung injury, with mild to severe diffuse alveolar damage (DAD) [124].
- High oxygen levels within 72 h after aneurysmal rupture is an uninfluenced predictor of cerebral vasospasm [125].
- In addition, liberal oxygen therapy increased 30-day mortality compared with conservative therapy [126].
- Controversial high-dose oxygen therapy recommendations to reduce surgical site infections (SSIs) by World Health Organization global guidelines for the prevention of surgical site infection [127].
- Hyperoxemia may reduce cardiac output and increase systemic vascular resistance in patients with cardiovascular failure [128].

### 6.3. Hyperthermia

Increased body temperature is frequently observed in patients following brain damage due to direct hypothalamic injury, cerebral inflammation or secondary infection indicating fever.

**Figure 2:**
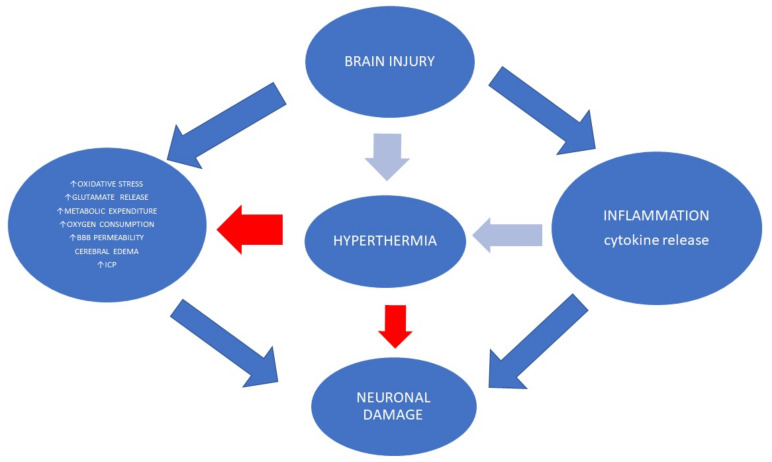
Hyperthermia is associated with poor neurological outcomes because it predisposes to greater secondary damage. Temperature changes lead to elevation of cytokine release, higher neutrophil activity and elevated metabolic expenditure. Hyperthermia also increases ROS generation and apoptosis.

Systemic hyperthermia is common after brain damage. In patients with brain injury, it is associated with poor neurological outcomes because it predisposes to worse secondary damage [129] (Figure 2).

It should be noted that hyperthermia is not always connected with fever. Fever is an adaptive reaction with, e.g., elevated neutrophil migration, activation of T-lymphocytes and increased interleukin-1 and interferon production [130]. Temperature changes lead to elevated cytokine release, higher neutrophil activity and elevated metabolic expenditure, elevated white blood cell accumulation, increased vascular permeability, and axonal damage [129]. Temperature changes also lead to cerebral blood flow conversion and hence cause changes to cerebral oxygenation. Recent animal research has documented that hyperthermia is associated with CD18 and intercellular adhesion molecule-1 (ICAM-1) activation, as well as with an increase in ionized calcium-binding adapter protein-1 (IBA-1) reactive microglia in the cortex [131]. Hyperthermia also increases ROS generation and apoptosis, for example by c-Jun N-terminal kinase (JNK) activation [132,133]. Wettervik et al. documented that hyperthermia leads to energy metabolism disturbances with no associations with higher ICP and lower CPP [134]. Importantly, higher temperature was connected with lower glucose concentration in cerebral tissue and a higher percentage of the lactate-pyruvate ratio >25 after 5 days [134]. In addition, the authors did not show a connection between hyperthermia and worse clinical outcomes.

The metabolic rate rises by around 20–25% during increased baseline core temperature of over 1.5–2 °C [135]. Recent data have shown that rising core temperature impacts increased cerebral glucose utilization and CMRO_2_ by 5 to 10% per degree Celsius [136]. Nunneley et al. observed that a temperature elevated by more than 2 °C is associated with a higher glucose metabolic rate in the hypothalamus, thalamus, corpus callosum, cingulate gyrus and cerebellum and lower in the caudate, putamen, insula and posterior cingulum [137]. In addition, an increase in brain metabolism by 10% following a 2 °C higher temperature may be connected with an important reduction of blood flow to support oxygenation [137]. Further, Spiotta et al. documented that hyperthermia did not reduce brain tissue oxygen [138]. It should be noted that higher body temperature leads to a better ability to maintain O_2_ uptake (VO_2_) because a higher fraction of the delivered O_2_ is extracted before the beginning of O_2_ supply subjection [139]. Cardiovascular adjustments, as well as sympathetic nerve activity during hyperthermia, also impact coupling between CMRO_2_ and CBF. The activity of sympathetic nerves increases under hyperthermia. Adrenergic nerves surround the vascular system, especially cerebral arteries [140]. Some authors suggest that vasoconstriction under hyperthermia causes decreased CBF [141]. However, there are a few doubts. First, in a hypermetabolic state under hyperthermia, different agents such as histamine, nitric oxide or prostanoids may counteract vasoconstriction [142]. Second, blood pressure significantly influences the cerebral vascular system. Third, the heterogenous response of cerebral vascularity may be modified by hyperthermia and changes in the density of alpha- and beta-adrenergic receptors [143]. Elevated body temperature impairs blood–brain barrier (BBB) integrity, especially with dehydration [144,145]. Finally, Bein et al. documented that the normalization of PaCO_2_ to eucapnia leads CBF to recuperate to a physiological state [18].

Clinical implications of hyperthermia:

- Systemic complications such as fever frequently occur in the early phase after brain damage and worsen secondary brain injury [134,146].
- Up to 50% of patients after acute brain injury experience fever during hospitalization [147].
- Brain temperature variations (>1 °C) are associated with poor functional outcomes [148].
- In sum, higher body temperature is associated with elevated metabolic demand and endogenous stress levels, blood pressure level changes, increases in cardiac output and heart rate, hyperventilation, the synaptic release of excitatory amino acids, increased ICP levels, ischemic cortical depolarizations, and BBB breakdown [146,149,150,151,152,153,154].
- Hyperthermia without oxygen delivery mismatch does not seem to induce significant neurochemical alterations such as glucose, lactate, pyruvate and glutamate levels [151].
- PbtO_2_ may be an important element to be monitored during a high body temperature episode to provide a view into oxygen metabolism in the brain [155].
- PbtO_2_ variations are observed under increased temperature increases in severe TBI patients. PbtO_2_ may rise on average in every third and decrease in every sixth episode of high temperature. Recent data have documented that the PbtO_2_ slope may occur simultaneously with CPP and MAP reduction [156].
- Temperature management to prevent fever is crucial for patients with severe traumatic brain injury. The international guidelines for severe brain injury highlight the importance of core temperature measurement and treatment above 38 °C [11,46].

### 6.4. Hypothermia

The main objective of current international clinical guidelines is to ameliorate final outcomes by inhibiting secondary injury, especially in the acute phase after damage. These protocols also include correction of temperature and therapeutic hypothermia. Moderate to deep hypothermia suppresses inflammation and decreases excitotoxicity and the production of free radicals, which is one of the mechanisms of neuroprotection [157]. Different levels of hypothermia improve neuronal tolerance to ischemia and inhibited neuronal death [158,159]. Cerebral hypothermia decreases ICP, maintains BBB function and ameliorates glucose utilization [160,161,162,163]. In addition, lower temperature suppresses hypoxic brain depolarization, releases neurotransmitters and decreases metabolism by protease activation and a high energy phosphate depletion rate [164,165]. Authors have even noted that deep hypothermia affects cerebral ATP production and improves survival after cardiac arrest by three to four times [164]. Importantly, hypothermia during ischemia reduces lipid peroxidation and essentially decreases ROS production [159,166]. Hypothermia reduces JNK activation and the apoptotic rate [132]. Hypothermia also activates a cascade of neuroinflammation and may improve M1/M2 macrophage polarization to a favorable phenotype [167].

Lower temperature improves cerebral metabolism after TBI and cerebral ischemia. In animal models, the metabolic rate for glucose (CMR_glc_) and CMRO_2_ is decreased, but significantly, ATP distribution is decreased more than synthesis is [168]. Furthermore, under a normoxic state, hypothermia decreases oxygen consumption in the brain as well as collateral depletion of CBF and delivery of oxygen (elevated cerebrovascular resistance and trace changes in oxygen extraction in the brain) [169]. Temperate hypoxia causes elevated CBF and oxygen extraction, followed by reduced cerebrovascular resistance [170]. Chihara et al., in an animal model of reduction in cerebral temperature by 1.6° ± 0.1° and hypoxia, documented that hypothermia results in decreased oxygen delivery, oxygen consumption and CBF. In addition, a significant improvement in cerebral vascular resistance is observed as well as no oxygen extraction shift [171]. Recent data by Hashem et al., using near-infrared spectroscopy (NIRS) and magnetic resonance imaging (MRI) methods, presented a significant decrease of CMRO_2_ in the cortex of around 37 and 32% of hypothermic mice and rats, respectively [172]. Therefore, targeting brain tissue oxygenation by different methods such as an NIRS device may be an important aspect of brain damage treatment guidelines for improving cerebral oxygenation, monitoring cerebrovascular reactivity (CVR) and final outcomes [173,174].

Clinical implications of hypothermia

- Therapeutic hypothermia is a crucial component of current clinical practice guidelines.
- Therapeutic hypothermia uses different cooling methods to maintain brain temperature at target levels.
- Therapeutic hypothermia improves neurological outcomes [175]. In contrast, accidental hypothermia at admission after TBI results in higher hospital mortality [176].
- Recently published data do not promote early prophylactic hypothermia within the first 6 h after damage in TBI patients [177].
- Body temperature of 35 to 35.5 °C after TBI reduces intracranial hypertension and preserves adequate CPP without cardiac dysfunction and oxygen debt [178]. In addition, hypothermia reduces high ICP [177].
- Recent meta-analyses have documented the importance of temperature measurement to avoid hypothermia in prehospital management [176].

## 7. Future Therapies

The oxygen-related mechanisms discussed have been a target for therapy in brain injuries. One crucial element in the management of patients with various forms of cerebral damage is the maintenance of oxygen homeostasis, supply and consumption, translating into normal mitochondrial metabolism. Both hypoxia and hyperoxia may present a negative effect on the final neurological outcome. Recent findings have shown the role of oxygen therapy in neuroprotection, related to normobaric hyperoxia (NBHO). Patients with acute brain injury treated with high oxygen levels (FiO_2_ 0.6–1.0) for two hours presented with improved redox balance and reduced lactate/pyruvate ratio (ΔLPR −3.07 *p* = 0.015) [179]. The NBHO method is based on continuous administration of oxygen in normal atmospheric pressure. Experimental data have documented the benefits of NBHO in ischemic stroke, hemorrhagic strokes and brain trauma [180,181].

Yang et al. in an animal model experimentally documented the effect of normobaric oxygen therapy (60%) on neurological functions, edema and HIF-1α, aquaporin 4 (AQP4) and Na+/H+ exchanger 1 (NHE1) expression (*p* < 0.05, respectively). These authors showed that therapy inhibits NHE1 expression and Na+ influx. These effects result in the reduction of brain edema following the movement of water by AQP4 [180]. Hyperbaric oxygen therapy (HBOT) is another therapy proposed in TBI. HBOT is 100% oxygen inhalation under a pressure greater than 1 absolute atmosphere. HBOT suppresses inflammation and defends BBB integrity and supports angiogenesis and neurogenesis [182,183]. Recent data in an animal model documented that oxygen therapy at an early stage after brain damage significantly decreased NF -κB and extracellular histones H1, H2A and H4 expression [184]. Histones are structural proteins in nuclei, an important factor in inflammation caused by hypoxia and ischemia [185]. In addition, HBOT inhibits the apoptotic mechanisms in neuronal cells and preserves the properties of mitochondrial membranes, reducing secondary damage [186,187]. Of course, the clinical effectiveness of HBOT is still controversial. Rockswold et al. documented in a small study that HBOT did not improve outcomes in a group of patients with closed head injury [188]. However, in other phase II clinical trials in 2013, Roackswold et al. demonstrated that combined HBOT with normobaric hyperoxia (NBHT) therapy improves oxidative metabolism and oxygen brain tissue partial pressure levels [189]. In addition, this therapy decreased intracranial hypertension, mortality and improved outcomes (measured by GOSE) [189]. Another study showed that HBOT significantly improved post-traumatic stress disorder symptoms, cognitive functions and decreased depression and anxiety [190].

The controversial effects of HBOT may be explained by the hyperoxic–hypoxic paradox (HHP). Recent research has shown that repetitive and periodic hyperoxia may induce molecular mechanisms and activate mediators similarly to hypoxia [191]. Activation of HIF, VEGF, SIRT, mitochondrial biogenesis and stem cell proliferation is observed during intermittent hyperoxia.

Another therapeutic option is the significant role of lactate in cerebral energy metabolism [192]. Experimental lactate supplementation in ischemic brain damage impacts decreased glutamate- and gamma-aminobutyric acid (GABA) release with improvement in electroencephalogram (EEG) [193]. Furthermore, Berthet et al. documented that lactate supplementation inhibits neuronal death in oxygen and glucose delivery disturbances [194]. The same treatment in middle cerebral artery occlusion and ischemia models also presents a significant neuroprotective effect [195,196]. Ichai et al., in randomized controlled trials, presented that hypertonic sodium lactate (HSL) treatment is more potent in reducing elevated ICP than mannitol in a group of TBI patients [197]. In addition, this effect lasts longer and is connected with improvement in jugular venous O_2_ saturation, glucose and lactate levels in plasma and pH. Patients also presented better neurological final outcomes [197]. The infusion of HSL for 3 h impacts extracellular metabolites. One theory is that these solutions contain metabolizable lactate and Na ions. Lactate in the brain induces an imbalance between anions and positive charges and counteracts the harmful cellular swelling by compensation of anion efflux [197] (Supplementary Materials).

Recent data have shown elevated lactate, pyruvate and glucose levels in the brain with associated lower glutamate and PbtO_2_ values as well as ICP. Bouzat et al. documented that these effects may be the result of a brain metabolism shift to elevated lactate utilization, sparing the effect of glucose. In addition, the inhibition of cerebral oxygenation may be secondary to alkalosis, which increases the affinity of oxygen to hemoglobin and suggests a beneficial effect [198]. In summary, hypertonic sodium lactate infusion reduces glutamate-related excitotoxicity, improves cerebral perfusion, buffers metabolic acidosis, decreases cerebral edema and ICP and improves cardiac performance [199,200,201].

## 8. Conclusions

Oxygen is crucial for the functionality of cerebral cells. Therefore, the mechanisms leading to disruption of oxygen supply and consumption are the subject of continuous intensive research. There is a growing need for novel therapeutic methods to reduce the cascade of pathological cellular processes.

## Figures and Tables

**Table 1:** Potentially effect of disorders in oxygen delivery to the brain on selected pathways and factors. Up arrows indicate the direction of the mechanism that may be intensified to varying degrees, depending on the causative factor. The number of arrows defines the intensity of the processes.

	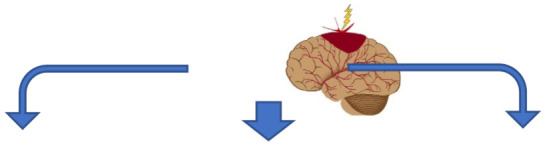		
	Hypoxia	Normoxia	Hyperoxia
Oxidative stress	↑↑	↑	↑↑↑
Hypoxia-inducible factor (HIF)	↑↑	↑	↑↑
Protein kinase B (Akt)	↑↑	↑	↑↑
Extracellular signal-regulated kinase (ERK)	↑↑	↑	↑↑
Brain-derived neurotrophic factor (BDNF)	↑↑	↑	↑↑
Erythropoietin (Epo)	↑↑	↑	↑↑
Neuroglobin (Ngb)	↑↑	↑	↑↑
Nitric oxide (NO)	↑↑	↑	↑↑

## Data Availability

Not applicable.

## Supplementary Materials

The following supporting information can be downloaded at: https://www.mdpi.com/article/10.3390/jpm12111763/s1, Table S1: Novel therapy approaches.
