# Supplementary material for: Cerebral Oxygen Delivery and Consumption in Brain-Injured Patients
Source: J Pers Med. 2022 Oct 25;12(11):1763. doi: 10.3390/jpm12111763 (PMC9698645; doi:10.3390/jpm12111763)
Supplement: Supplementary file 1 [file jpm-12-01763-s001.zip › jpm-1911887-supplementary.pdf]

Table S1. Novel therapy approaches. CMD- Cerebral microdialysis; LPR -lactate to pyruvate ratio; BWC-brain water content; TJ -tight junctions; MMP- matrix metalloproteinase; BBB- blood brain barrier; TBI- traumatic brain injury.

	YEAR	MATERIAL	N	METHODS	EFFECT
**NORMOBARIC-HYPEROXIA (NBHO)**					
Ghosh et al [179].	2016	TBI patients	16	20-min normobaric hyperoxia challenge in the acute phase (24–72 h) post-injury	-increased $p_{br}O_2$<br>- LPR reduction<br>-increased cytochrome *c* oxidase (CCO) oxidation
Yang et al [180].	2019	Male rats	150	60% NBHO; and 100% NBHO groups	-reduction of neurological impairment, all BWC and decreased the infarct volume<br>-supression of NHE1 expression, which decrease Na+ influx, resulting in the movement of water through AQP4 to reduce brain edema.
Liang et al [181].	2015	Male rats	144	100% NBHO for 30minutes	- slower MMP-9 Induction<br>-loss of TJ in ischemic microvessels<br>- slows down the deterioration of BBB
**HYPERBARIC OXYGEN THERAPY (HBOT)**					
Liang et al [184].	2020	Male and female rats (TBI)	120	95% oxygen in pressure was increased to 1.6 and 2.2 atm over 10 min respectively	1.6ATA HBO has an important protective role in inhibiting both the cytotoxic and the pro-inflammatory actions of histones in the early stage of secondary brain injury.
Palzur et al [186].	2004	Rats	25	hyperbaric 100% oxygen therapy with 150-L pressure chamber 3h after injury, twice every day for 3 days and normobaric therapy	-Lesion surface decreased
Palzur et al [187].	2008	Male rats (cortical deformation)		100% oxygen at 2.8 ATA during two sessions of 45 minutes	- reduction of caspases 3 and 9 activation in HBOT treated animals<br>- preserved mitochondrial integrity

					- reduction of the mitochondrial pathway of apoptosis
Rockswold et al [189].	2013	TBI patients	42	combined HBO2/NBH (60 minutes of HBO2 at 1.5 atmospheres absolute [ATA] followed by NBH, 3 hours of 100% fraction of inspired oxygen [FiO2] at 1.0 ATA	-increased of brain tissue partial pressure of O2<br>-decreased microdialysate lactate/pyruvate ratios<br>-lower intracranial pressure values<br>-lower microdialysate glycerol<br>-reduction of mortality
Harch et al [190].	2020	Mild TBI patients	63	150 kPa/60 minutes, once daily, 5 days per week in 8 weeks	-significant improvements in postconcussion symptoms,<br>- improvement of cognitive variables (memory, cognition/speed of information processing), and behavioral/emotional problems (anxiety, depression, PTSD symptoms, sleep, and quality of life)
**LACTATE SUPPLEMENTATION**					
Phillis et al [193].	1999	Rats with cerebral ischemia/reperfusion injury	30	sodium lactate 20 or 40mM	-reduced ischemia-evoked amino acid effux<br>-enhanced EEG rocovery during reperfusion
Berthet et al [194].	2009	Male mice	26+13	intracerebroventricular injection of 2 μL of 100 mmol/L l-lactate, immediately after reperfusion	-decreased of lesion volume<br>-improvement of neurological outcome (A later injection 1 h after reperfusion did not reduce lesion size, but significantly improved neurologic outcome)
Berthet et al [196].	2012	rats	70	Na L-lactate was administered intracerebroventricularly or intravenously at reperfusion	-smaller neurological deficits at 7 days and 14 days after ischaemia<br>- attenuated hemispheric atrophy at 14 days
Ichai et al [197].	2009	TBI patients in ICU	34	receive equally hyperosmolar and isovolumic therapy, consisting of either mannitol or sodium lactate	Compared to mannitol, the effect of the lactate solution on ICP was significantly more pronounced (p = 0.016), more prolonged (p= 0.009) and more frequently successful (p=0.053)

Bouzat et al [198].	2014	TBI patients	15	3-h intravenous infusion of hypertonic sodium lactate (aiming to increase systemic lactate to ca. 5 mmol/L), administered in the early phase following TBI.	-significant increase in CMD concentrations of lactate, pyruvate, and glucose (all $p < 0.01$)<br>-concomitant reduction of CMD glutamate ($p = 0.06$) and ICP ($p < 0.01$)
Ros et al [200].	2001	rats	36	infusion of sodium lactate, sodium glutamate and D-lactic acids by probe below the dura	-<br>-addition of L- lactate, but not D-lactate, reduces the size of the lesion and alters the metabolic effects of an infusion of 100 mM glutamate<br>- neuroprotective effect of lactate (lowered pH on NMDA receptors and lactate as a metabolic substrate)
